# Experimental investigation on *n*-butanol/methyl oleate dual fuel RCCI combustion in a single cylinder engine at high-load condition

**DOI:** 10.1038/s41598-021-03693-y

**Published:** 2021-12-20

**Authors:** Xin Wang, Qian Zhang, Fangjie Liu, Yifan Jin, Xin Li

**Affiliations:** College of Vehicle and Traffic Engineering, Henan University of Science and Technology, Luoyang, 471003 China

**Keywords:** Environmental sciences, Energy science and technology

## Abstract

Reactivity controlled compression ignition (RCCI) engines have a high thermal efficiency as well as low emissions of soot and nitrogen oxides (NOx). However, there is a conflict between combustion stability and harmful emissions at high engine load. Therefore, this work presented a novel approach for regulating *n*-butanol/methyl oleate dual fuel RCCI at high engine load in attaining lower pollutant emissions while maintaining stable combustion and avoiding excessive in-cylinder pressure. The tests were conducted on a single cylinder engine under rated speed and 90% full load. In this study, *n*-butanol was selected as a low-reactivity fuel for port injection, and *n*-butanol/methyl oleate blended fuel was used for in-cylinder direct injection. Combustion and emission characteristics of the engine were first investigated with varied ratios of *n*-butanol port injection (PFI) and direct injection (DI). Results showed that as the ratio of *n*-butanol PFI and DI rose, the peak cylinder pressure and heat release rate increased, while NOx and soot emissions reduced, and carbon monoxide (CO) and hydrocarbon (HC) emissions increased under most test conditions. When R_NBPI_ = 40% and R_NBDI_ = 20%, the soot and NOx emissions of the engine were near the lowest values of all test conditions, yet the peak in-cylinder pressure and fuel consumption could not increase significantly. Therefore, the possibility of optimizing the combustion process and lowering emissions by adjusting the pilot injection strategy was investigated utilizing these fuel injection ratios. The results revealed that with an appropriate pilot injection ratio and interval, the peak in-cylinder pressure and NOx emission were definitely reduced, while soot, CO, and HC emissions did not significantly increase.

## Introduction

Diesel engines are extensively utilized in transportation vehicles and construction machinery. However, the compression ignition combustion mode of the conventional diesel engine results in higher NOx and soot emissions. These two pollutants could be reduced by lowering the combustion temperature and raising the premixed combustion ratio as much as possible^1^. As a consequence, innovative combustion modes such as homogeneous charge compression ignition (HCCI)^2^, premixed charge compression ignition (PCCI)^3,4^, partially premixed combustion (PPC)^5^, and RCCI^6^ have been proposed. The RCCI combustion mode is characterized by the presence of two distinct fuel supply systems. A port injection fuel system injects volatile and low-reactive fuels such as gasoline, methanol, and ethanol into the inlet to produce a homogeneous mixture. The second kind of fuel system is in-cylinder direct injection, which directly injects highly reactive fuels like diesel and biodiesel into the cylinder. By adjusting the ratio of port injection and direct injection fuel in the cylinder, the response activity of the mixture in the cylinder could be changed to control the combustion phase under different load conditions. Compared to other combustion modes, the RCCI mode enables more precise control of the ignition and combustion processes^7–10^.

The RCCI combustion mode has been extensively investigated since it was proposed. The researchers selected gasoline^11^, natural gas^12^, methanol^13^, ethanol^14^, *n*-butanol^14–16^, hydrogen^17^ as low-reactivity fuels for port injection. The most often utilized highly active fuels for direct injection into the cylinder were diesel^18,19^ and biodiesel^20–24^. According to the research findings, as compared to traditional compression ignition, the RCCI combustion mode significantly reduced soot emissions while increasing CO and HC emissions, and the NOx emissions change trend was related to engine load and port injection ratio.

Throughout the engine's life, carbon dioxide (CO_2_) emissions could be reduced by fueling it with renewable biofuels. Currently, biofuels such as alcohols^25^, biodiesel, and furans^26–29^ were used. *n*-Butanol has a lower vapor pressure and a higher flash point than methanol and ethanol, making it potentially safer to supply and use. It is less corrosive, which prolongs the life of the fuel system. When utilized in PPC, HCCI, and RCCI combustion modes, *n*-butanol is less prone to cause misfires due to its higher cetane number than methanol and ethanol. Varol^30^ and Li^31^ investigated the combustion and emission characteristics of gasoline blended with methanol, ethanol, and *n*-butanol on a spark ignition engine. Results revealed that gasoline mixed with *n*-butanol had the lowest fuel consumption because the heating value of *n*-butanol was greater than that of ethanol and methanol. Biodiesel is physically and chemically similar to diesel. Since the carbon–oxygen double bond in the ester group does not break during combustion or pyrolysis^32^, the quantity of carbon atoms converted to soot precursors is reduced, resulting in a decrease in soot emissions. Liu et al.^33^ investigated the RCCI combustion and emission characteristics of *n*-butanol through port injection (referred to as PFI) and biodiesel via in-cylinder direct injection (referred to as DI). By adjusting the *n*-butanol injection ratio and EGR rate, it was possible to achieve 76% of full load while maintaining acceptable NOx and soot emissions. Zheng et al.^14,15^ discussed the distinctions between RCCI and blended combustion using biofuel. The blended fuel mode had a higher thermal efficiency. By contrast, when the ratio of *n*-butanol PFI rose, the thermal efficiency of the RCCI mode decreased. CO and HC emissions of the RCCI combustion mode rose as the ratio of *n*-butanol PFI increased, whereas the CO and HC emissions of the blended mode were less sensitive.

Biodiesel is a mixture of multiple fatty acid methyl esters (FAMEs). Although most biodiesel consists of the same FAMEs, the proportion of each FAME varies due to biodiesel derived from various feedstocks^34,35^. Various kinds of biodiesel include a sizable proportion of methyl oleate^36,37^. For instance, methyl oleate accounted for 60% of rapeseed biodiesel. In order to simplify the combustion analysis as well as assure the consistency of the experimental outcomes, methyl oleate was used for this study. El-Seesy et al.^38^ compared the combustion and emission characteristics of diesel engines fueled with diesel and methyl oleate, finding that when methyl oleate was used, the peak pressure of the diesel engine was reduced by 4%, while soot and NOx emissions were reduced by 2% and 6%, respectively, and the brake specific fuel consumptions (BSFCs) was increased by 5%. In comparison to gas oil, Myo et al.^39^ demonstrated that using methyl oleate effectively decreased peak pressure at full load in a single cylinder DI diesel engine, while also significantly reducing HC and soot emissions, marginally lowering CO emissions, and slightly increasing NOx emissions at 25–100% engine load conditions. Cui et al.^40^ found that adding 40% methyl oleate by volume to diesel reduced particle number (PN) at high engine speeds owing to the oxygen concentration in the methyl oleate molecule, and particle diameter (Pd) was smaller under all conditions. Soloiu et al.^41^ studied the RCCI combustion of methyl oleate DI and *n*-butanol PFI. The dual-fuel RCCI combustion mode significantly lowered NOx and soot emissions compared to traditional in-cylinder direct injection of methyl oleate, while CO and HC emissions increased at indicated mean effective pressures (IMEP) of 4 and 5 bar.

However, when the RCCI combustion mode is extended to high loads, a conflict arose between combustion stability and hazardous emission control^42,43^. To simultaneously lower NOx and soot emissions, it is required to increase the EGR rate and the port injection ratio, which could easily result in an overly high pressure rise rate.

Therefore, this work presented a novel approach for regulating *n*-butanol/methyl oleate dual fuel RCCI at a high engine load. That is, a specified quantity of *n*-butanol was used for the port injection, the blended fuel of *n*-butanol and methyl oleate is injected directly into the cylinder. This fuel injection approach could effectively promote the evaporation of in-cylinder direct injection fuel by utilizing the micro-explosion effect of *n*-butanol^44,45^. It was feasible to lower pollutant emissions while maintaining stable combustion and avoiding excessive in-cylinder pressure by optimizing the fuel injection strategy.

## Experimental setup and methodology

### Experimental setup

**Figure 1:**
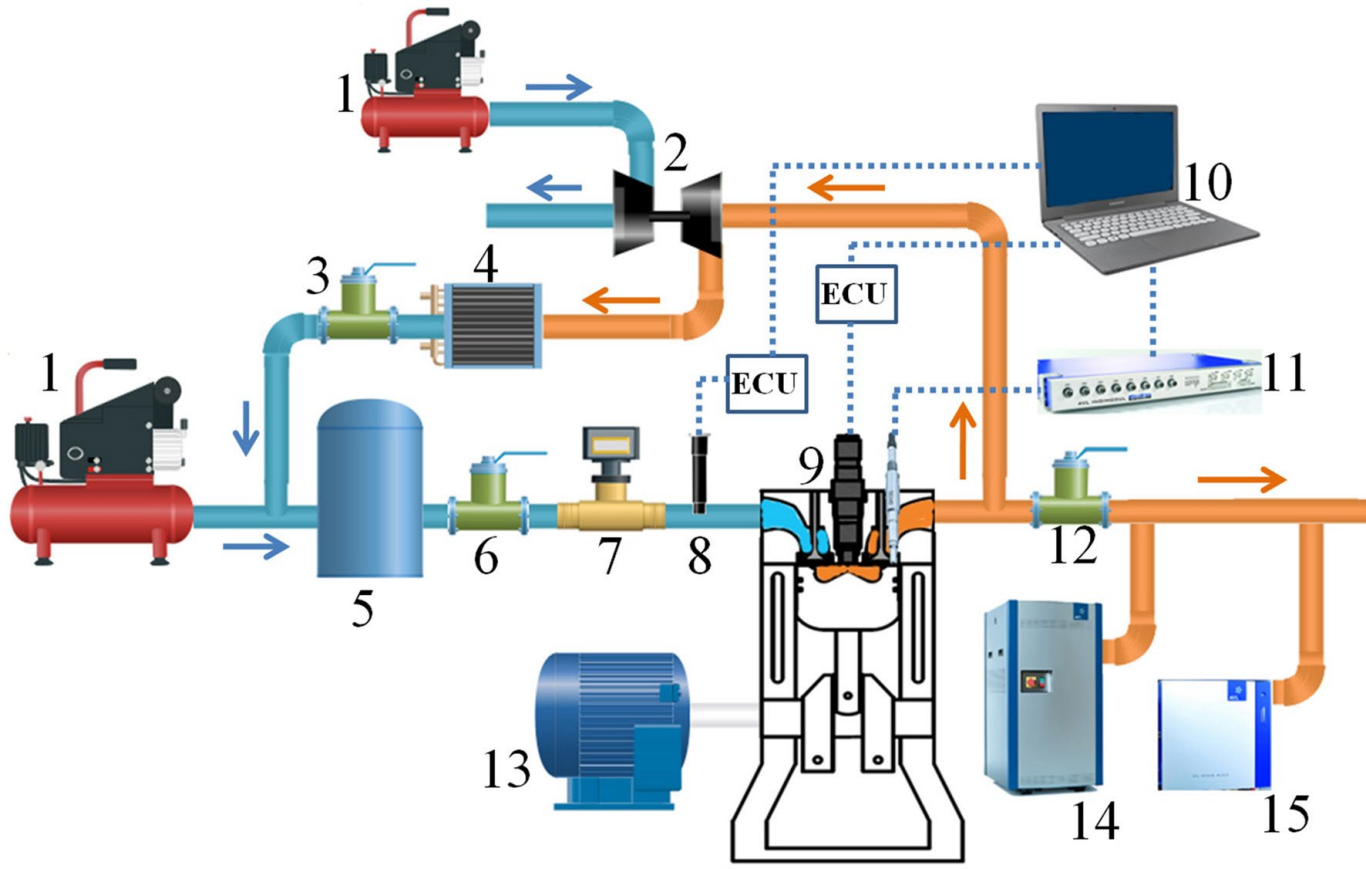
Schematic diagram of the experimental setup. 1. Gas compressor; 2. Supercharger; 3. EGR valve; 4. EGR cooler; 5. Buffer tank; 6. Inlet control valves; 7. Air flow meter; 8. PFI injector; 9. DI injector; 10. Computer; 11. Combustion analyzer; 12. Exhaust back pressure valve; 13. Dynamometer; 14. Gas analyzer; 15. Smoke meter.

The *n*-butanol/methyl oleate dual-fuel RCCI combustion experiment was carried out on a single-cylinder four-stroke compression ignition engine. The engine was a single-cylinder version of a multi-cylinder marine diesel engine designed mainly for scientific research. The technical specifications of the test engine are shown in Table 1, and the schematic of the experimental setup is depicted in Fig. 1. The compressor boosted the intake pressure to match that of a multi-cylinder marine diesel engine. The backpressure valve throttled the exhaust flow and was intended to replicate the exhaust backpressure. Due of the high intake pressure of the single-cylinder engine, it was difficult to feed EGR gas into the intake pipe. Therefore, the compressor fed high-pressure air to a turbocharger, which increased the pressure of the EGR gas before it entered the intake pipe. The target EGR ratio was achieved by altering the opening of the EGR valve. Fresh intake air and EGR gas were thoroughly mixed in the surge tank. An *n*-butanol injection system assembly was mounted on the intake pipe. The *n*-butanol/methyl oleate blend fuel was injected via the common rail system of the single-cylinder engine. The cylinder pressure and heat release rate were determined using a comprehensive set of combustion measuring equipment from the AVL company, including pressure sensors, charge amplifiers, data acquisition, and indicating software. The AVL AMA i60 exhaust measuring system was used to determine gas emissions, and the AVL 415S smoke meter was used to measure smoke emissions. Table 2 shows detailed information about the measuring equipment, including their measuring range, resolution, and uncertainty.

**Table 1:** Engine specifications.

Items	Specifications
Number of cylinders	1
Power output @ 1800 rpm (kW)	125
Max. torque @ 1350 rpm (N·m)	730
Combustion chamber shape	Stepped-lip
Bore diameter (mm)	170
Stroke length (mm)	195
Connecting rod length (mm)	350
Displacement (L)	4.426
Compression ratio	13.5:1
PFI pressure (MPa)	0.3
DI pressure (MPa)	150
Inlet valve close	− 139.5° CA ATDC
Exhaust valve open	117.5° CA ATDC

**Table 2:** Details of measuring devices.

Measured parameter	Device	Measuring range	Resolution	Uncertainty
Exhaust concentration	AVL AMA i60	0–100%	1 ppm	≤ 0.5% of measured value
Smoke meter	AVL 415S	0–10 FSN	0.001FSN	0.005 + 3% of measured value FSN
Cylinder pressure	AVL GH15D	0–250 bar	19 pC/bar	± 0.3 bar
Fuel mass flow rate	AVL 7355	0–125 kg/h	0.01 kg/h	≤ 1% of measured value
Air flow meter	ToCeiL20N100	0–1200 kg/h	–	≤ 1% of measured value

### Test fuels

In this investigation, *n*-butanol was selected as a low-reactivity fuel for port injection, and an *n*-butanol/methyl oleate blended fuel was used for in-cylinder direct injection. *n*-Butanol [CH3(CH2)3OH] (99%, Tianjin Kemiou Chemical Reagent Co., Ltd.) and methyl oleate [CH3(CH2)7CHCH(CH2)7COOCH3] (99%, Jinan Dehou Chemicals Co., Ltd.) were provided commercially. Table 3 provides the physical and chemical properties of *n*-butanol and methyl oleate, as well as those of diesel for comparison.

**Table 3:** Fuel properties^15,36^.

Properties	*n*-Butanol	Methyl oleate	Diesel
Molecular formula	C4H10O	C19H36O2	C10-C20
Molecular weight	74.1	296.5	190–220
Oxygen content (wt%)	21.6	10.8	–
Density (g/mL^3^) at 20 °C	0.809	0.874	0.83–0.85
Cetane number	17–25	56	52–55
Viscosity at 40 °C (mm^2^/s)	2.22	4.51	3.35
Lower heating value (MJ/kg)	33.2	37.1	42.8
Enthalpy of vaporization at 20 °C (kJ/kg)	582	285	270
Boiling point (°C)	117	382	180–370

### Experimental methodology

When the engine was under heavy load, a high ratio of *n*-butanol PFI resulted in excessive peak pressure in the engine cylinder. Thus, the ratio of *n*-butanol PFI (represented by R_NBPI_) was 20–60% of the total heating value of fuel injection, while the ratio of *n*-butanol DI (represented by R_NBDI_) was between 0–30%.

R_NBPI_ and R_NBDI_ are expressed using the following equations:

1 $$R_{NBPI} = \frac{{m_{NBPI} \times 33.2}}{{m_{NBPI} \times 33.2 + m_{NBDI} \times 33.2 + m_{MO} \times 37.1}}$$

2 $$R_{NBDI} = \frac{{m_{NBPI} \times 33.2}}{{m_{NBDI} \times 33.2 + m_{MO} \times 37.1}}$$

where m_NBPI_ is the mass of *n*-butanol PFI, m_NBDI_ is the mass of *n*-butanol DI, m_MO_ is the mass of methyl oleate DI.

The engine was operated at its rated speed (1800 r/min) with a load of 103.5 kW (corresponding to 90% of maximum engine out power). The engine was not tested at full load to avoid exceeding the engine's permitted value for cylinder pressure when the *n*-butanol PFI ratio is too high. The EGR rate was 20%. To maintain high thermal efficiency for the diesel engine, the injection timing of the direct injection fuel was adjusted in real time in each test case to maintain the CA50 (crankshaft angle corresponding to 50% accumulative heat release rate) at 10° CA ATDC. The heating value of the fuel delivered to the engine stayed constant during each cycle. To begin, the engine ran at 1800 rpm and 103.5 kW when fueled with pure methyl oleate in-cylinder direct injection. The consumption of methyl oleate at this moment was then recorded. Thus, the quantity of methyl oleate, *n*-butanol PFI and DI required for each cycle was calculated according to the definition of Eqs. (1) and (2).

The experiment was conducted in two phases. To begin, the combustion and emission characteristics of the engine fueled with various ratios of *n*-butanol PFI and DI were investigated in order to determine the ideal injection ratios with superior engine performance and emissions. Following that, various pilot-main injection strategies were investigated based on these optimum injection ratios to further optimize combustion and reduce emissions.

## Results and discussions

### Combustion and emission characteristics of the engine with varied ratios of *n*-butanol PFI and DI

The performance of the engine with R_NBPI_ = 20%, 40%, 60% and R_NBPI_ = 0, 10%, 20%, 30% was initially investigated. The operating settings of the engine are shown in Table 4.

**Table 4:** Experimental conditions.

Items	Set value
Engine speed (rpm)	1800
EGR rate (%)	20
*n*-Butanol PFI ratios (%)	20, 40, 60
*n*-Butanol DI ratios (%)	0, 10, 20, 30
Intake temperature (°C)	40
Intake pressure (MPa)	0.3

**Figure 2:**
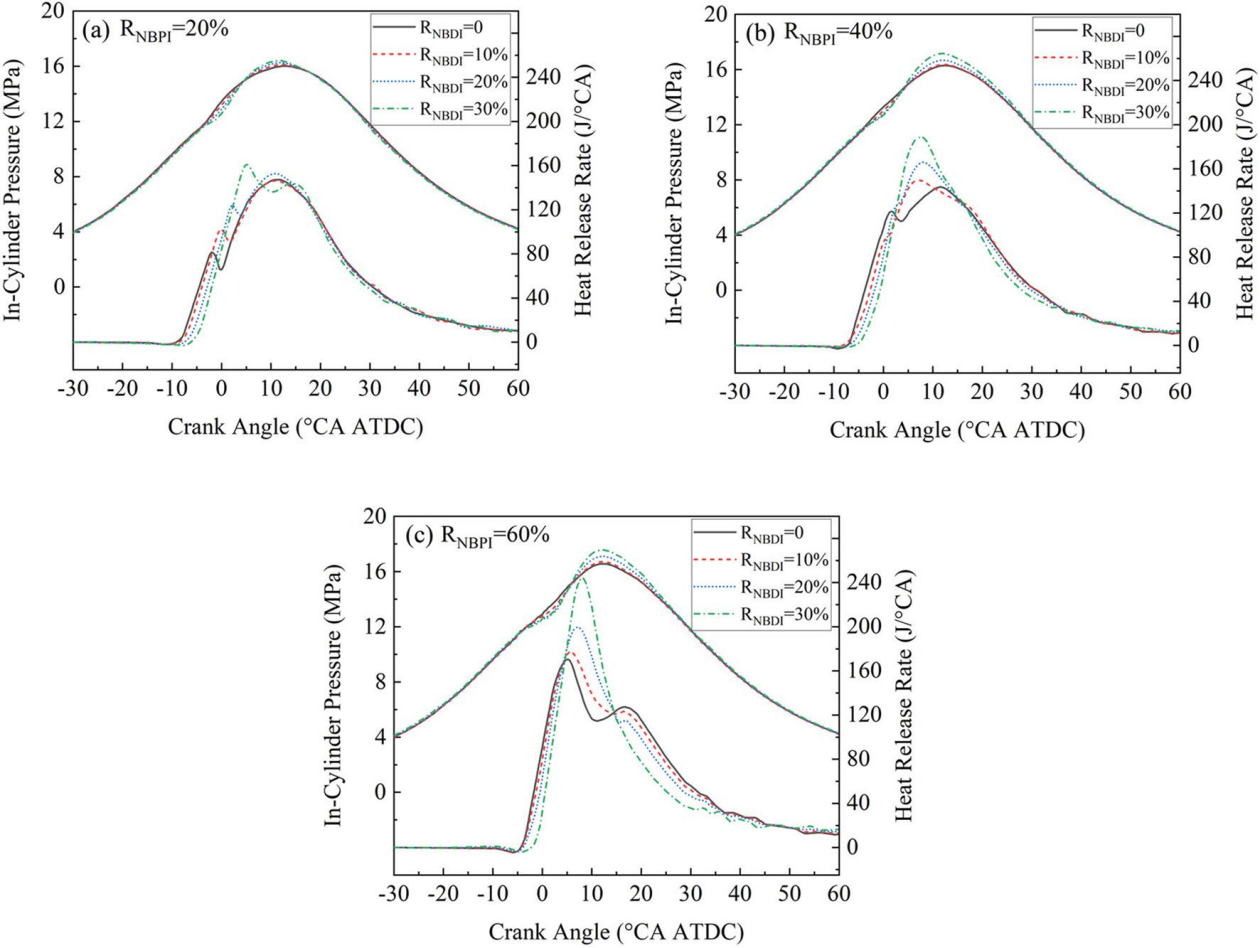
In-cylinder pressures and heat release rates of various R_NBPI_ and R_NBDI_.

The comparison of cylinder pressures and heat release rates for various fuel injection strategies is shown in Fig. 2. In general, as the ratio of *n*-butanol PFI and DI rose, the peak cylinder pressure increased progressively. When R_NBPI_ = 20% and R_NBDI_ = 0, the peak cylinder pressure was 16.02 MPa, as R_NBDI_ grew to 30%, the peak cylinder pressure rose to 16.41 MPa. For R_NBDI_ = 40%, the peak cylinder pressure rose from 16.29 to 17.17 MPa as the R_NBDI_ increased from 0 to 30%. For R_NBDI_ = 60% and R_NBDI_ = 0, the peak cylinder pressure was 16.57 MPa. The highest peak cylinder pressure (17.57 MPa) occurred when R_NBPI_ = 60% and R_NBDI_ = 30%, which elevated the lowest peak cylinder pressure (16.02 MPa) by 9.7%. The methyl oleate was directly injected into the cylinder, where it atomized and evaporated. When the working medium in the cylinder reached a specified temperature and pressure, the methyl oleate induced spontaneous combustion and then ignited the *n*-butanol. The ignition delay time was prolonged as the ratio of *n*-butanol PFI and DI increased. During the ignition delay period, a larger combustible mixture was generated, resulting in a greater heat release of premixed combustion.

It can also be noticed that, as the ratio of *n*-butanol PFI and DI rose, the heat release rate increased progressively. In proportion to the rise in R_NBDI_, the peak of premixed exotherm gradually increased, as did the amount of premixed heat released. For R_NBPI_ = 60% and R_NBDI_ = 30%, the heat release rate exhibited a single peak. The reason for this was that the quantity of methyl oleate injected was lowered, the amount of active free radicals produced by the low-temperature reactions of methyl oleate dropped, and the activity of the in-cylinder medium decreased, resulting in a prolonged ignition delay time. Additionally, the *n*-butanol injected into the cylinder promoted the atomization of methyl oleate, resulting in the complete mixing of methyl oleate with the *n*-butanol in the cylinder. When methyl oleate was self-ignited, it simultaneously ignited the *n*-butanol. As a consequence, only one heat release process with a high peak heat release rate existed.

**Figure 3:**
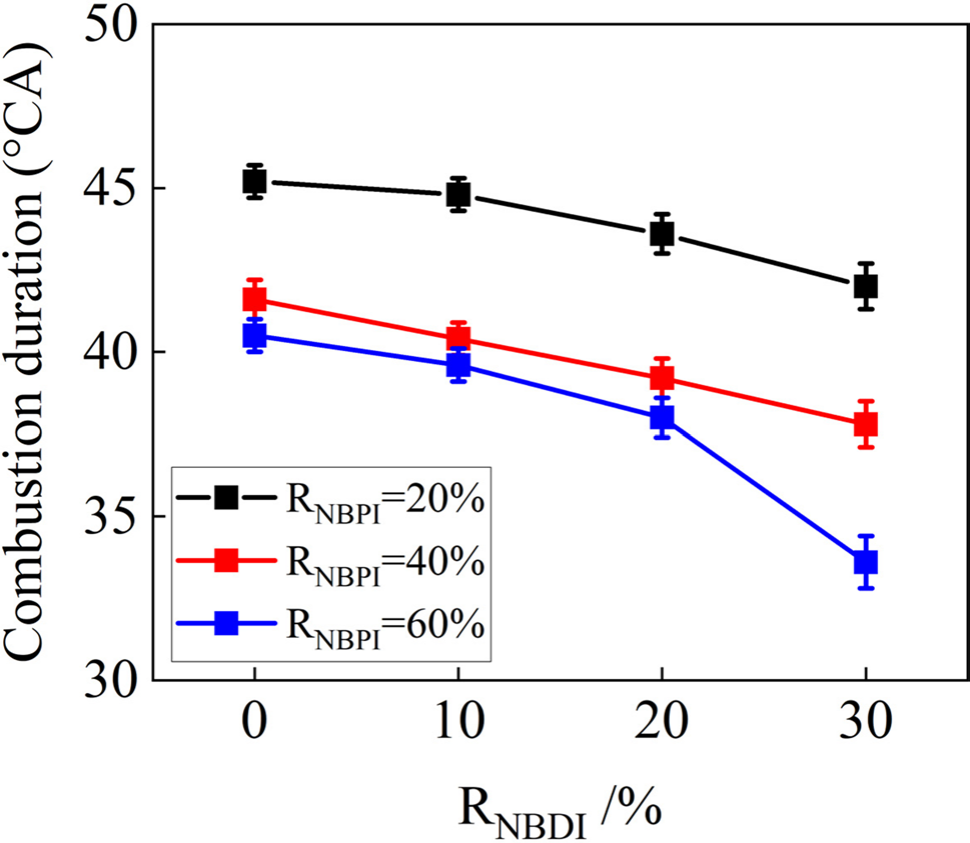
Combustion durations of various R_NBPI_ and R_NBDI_.

Figure 3 compares the combustion durations for various fuel injection strategies. As can be observed, when R_NBPI_ = 40% and 60%, the combustion duration was considerably shorter than when R_NBPI_ = 20%. This was because when the ratio of *n*-butanol PFI rose, the quantity of methyl oleate DI dropped, resulting in a greater volume of combustible mixture with a longer ignition delay time. When methyl oleate was self-ignited, it accelerated the combustion of a large number of premixed fuels, which led to a shorter combustion duration. The combustion duration gradually decreased with increasing R_NBPI_ for the same R_NBDI_. This was due to the fact that as the amount of *n*-butanol DI increased, the cetane number of the blended fuel decreased, causing the ignition delay time to lengthen, which increased the proportion of premixed combustion. The premixed combustion rate was faster than diffusion combustion, resulting in a shorter combustion duration. When RNBPI = 60%, the combustion duration with R_NBDI_ = 30% is significantly lower than that with RNBDI = 20%. This was owing to the lengthy ignition delay time with a modest amount of methyl oleate, which enabled the methyl oleate to adequately atomize and evaporate in the cylinder. As demonstrated in Fig. 2c, the consumption of methyl oleate was predominantly premixed combustion with a fast combustion rate, resulting in a very short combustion duration.

**Figure 4:**
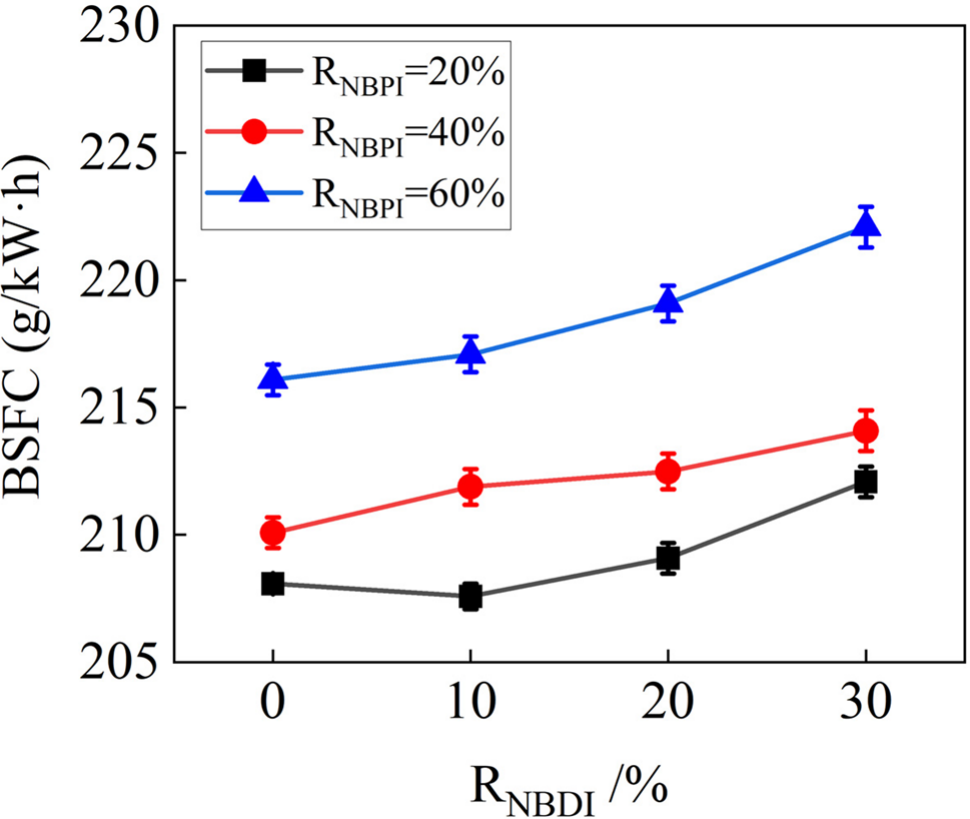
BSFCs of various R_NBPI_ and R_NBDI_.

The BSFCs for various fuel injection strategies are shown in Fig. 4. It was obvious that as the increment of R_NBPI_, the BSFC gradually increased. Because *n*-butanol has a lower heating value than methyl oleate, a higher R_NBPI_ needed more fuel injection to achieve the same power output. When R_NBPI_ = 20% and R_NBDI_ = 10%, the BSFC dropped slightly. This was because the *n*-butanol DI caused "micro explosion" due to its low boiling point, promoting the atomization of methyl oleate. Thereby the combustion efficiency was improved. However, with a further increment of R_NBDI_, BSFC progressively rose. When R_NBPI_ = 40% and 60%, the BSFC rose as the R_NBDI_ increased. This was also due to the fact that a higher R_NBDI_ resulted in a lower heating value per unit mass of fuel for direct in-cylinder injection, which led to a higher rate of fuel consumption.

**Figure 5:**
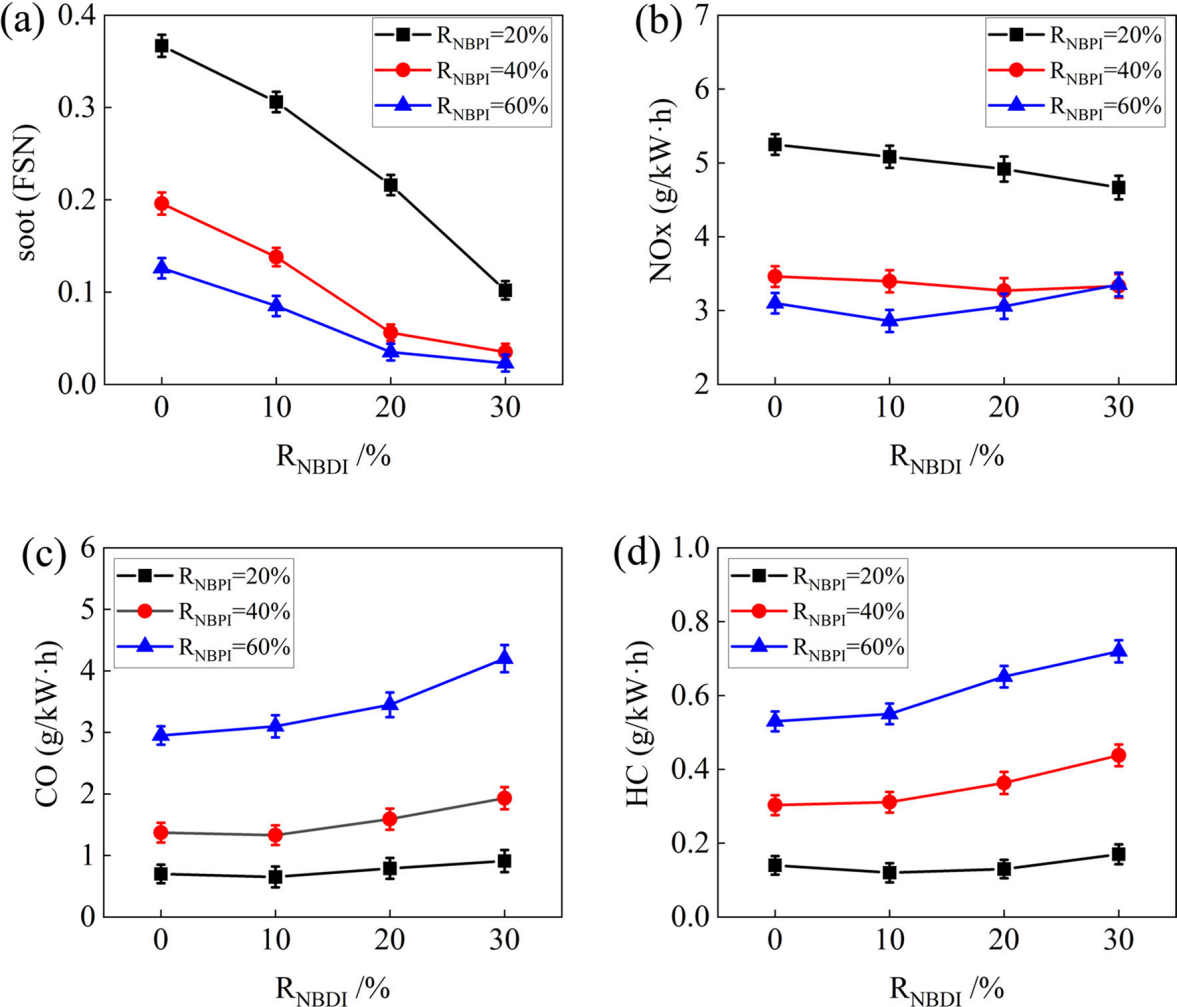
Emissions of various R_NBPI_ and R_NBDI_.

Figure 5 illustrates the variation in emissions caused by different fuel injection strategies. As shown in Fig. 5a, soot emissions decreased as the ratio of *n*-butanol PFI rose. The reason was that with the increment of the *n*-butanol PFI ratio, the quantity of methyl oleate DI in the cylinder dropped. The area of the excessively rich mixture shrunk, resulting in a reduction in soot precursor production. When R_NBPI_ = 40% and 60%, soot emissions were considerably lower than R_NBPI_ = 20%. At the same R_NBPI_, soot emissions decreased as the ratio of *n*-butanol DI rose. This was because, on the one hand, the injection quantity of methyl oleate was reduced. On the other hand, the "micro explosion" action of *n*-butanol DI promoted the atomization of methyl oleate. When R_NBPI_ = 40% and 60%, and R_NBDI_ = 20%, the soot emissions were already relatively low. As R_NBDI_ increased from 20 to 30%, the reduction in soot emission became less noteworthy. This occurred because when the R_NBDI_ reached 20%, the methyl oleate may already be atomized more completely owing to the micro-explosion action of *n*-butanol DI, and its diffusion combustion was substantially decreased, resulting in extremely low soot emission. Even though the R_NBDI_ was increased to 30%, the atomization of methyl oleate remained adequate, just as it had been when the R_NBDI_ was 20%. As a consequence, the potential of further lowering soot emissions was not especially apparent. In addition, it can be observed in the figure that the test cases with extremely low soot emissions were R_NBPI_ = 40%, R_NBDI_ = 20% and 30%, and R_NBPI_ = 60%, R_NBDI_ = 20% and 30%. Compared to R_NBPI_ = 20% and R_NBDI_ = 0, soot emissions were decreased by 84.7% when R_NBPI_ = 40% and R_NBDI_ = 20%, and by 93.7% when R_NBPI_ = 60% and R_NBDI_ = 30%.

As shown in Fig. 5b, NOx emissions decreased as the ratio of *n*-butanol PFI rose. This was due to the fact that as the methyl oleate DI ratio decreased, the region of the overly rich methyl oleate mixture diminished, thus reducing high temperature area in the cylinder and resulting in lower NOx emissions. For R_NBPI_ = 40% and 60%, NOx emissions were much lower than R_NBPI_ = 20%. A reduction in methyl oleate injection into the combustion chamber was the primary reason for this. On the other hand, a longer ignition delay period allowed the methyl oleate to fully atomize and evaporate, thereby reducing the high temperature area in the cylinder. When R_NBPI_ = 20%, NOx emissions decreased as the ratio of *n*-butanol DI rose. This was because *n*-butanol DI has a high latent heat of evaporation, which was beneficial to reducing the temperature in the cylinder. When R_NBPI_ = 40% and 60%, there was no discernible change in NOx emissions. The *n*-butanol DI lowered the temperature in the cylinder. It did, however, simultaneously introduce a specific quantity of O atoms. The final NOx emissions were the consequence of the combined effects of temperature, the duration of the combustion heat release, and the quantity of O atoms.

CO emissions increased dramatically as the ratio of *n*-butanol PFI rose, as seen in Fig. 5c. Compared to R_NBPI_ = 20%, CO emissions increased more than doubled when R_NBPI_ = 40% and three to four times when R_NBPI_ = 60%. This was because as the ratio of *n*-butanol PFI increased, the combustion duration was shortened, and when the cylinder temperature dropped, the CO generated during the *n*-butanol combustion process could not be further oxidized to CO_2_. When R_NBPI_ = 20% and 40%, and R_NBDI_ = 10%, CO emissions decreased slightly. This was due to a tiny quantity of *n*-butanol DI enhanced methyl oleate evaporation, which improved combustion efficiency. CO emissions steadily increased when the ratio of *n*-butanol PFI was raised further. Because of an increase in the quantity of *n*-butanol in the cylinder that had not been completely oxidized. Continued growth in R_NBDI_ resulted in a progressive increase in CO emissions. Because of the high R_NBDI_, the fuel direct injection into the cylinder atomized more completely, leading to a low combustion temperature; on the other hand, the shorter combustion duration resulted in an increase in the amount of CO that could not oxidize to CO_2_. When R_NBPI_ = 60%, CO emissions increased in proportion to the *n*-butanol DI ratio.

Figure 5d illustrates a significant rise in HC emissions when the *n*-butanol PFI ratio is raised. The reason for this was that as the ratio of *n*-butanol PFI increased, the *n*-butanol in the cylinder was quenched during the combustion process, increasing the quantity of *n*-butanol stored in the slit. When the piston went down, this portion of the *n*-butanol fails to completely oxidize, resulting in higher HC emissions. Furthermore, the *n*-butanol concentration at the top surface of the piston and the cylinder wall surface is high due to the increased port injection ratios, and quenching occurs when the flame propagates to the cold wall surface, leading to higher HC emissions. For R_NBPI_ = 20%, the ratio of *n*-butanol DI had minimal effect on HC emission. While for R_NBPI_ = 40% and 60%, HC emissions increased dramatically with an increase in R_NBDI_.

### Effect of pilot injection strategies on combustion and emission characteristics

According to the analysis in “Combustion and emission characteristics of the engine with varied ratios of *n*-butanol PFI and DI”, with R_NBPI_ = 40% and R_NBDI_ = 20%, the engine's soot and NOx emissions were near the lowest of all test instances. Simultaneously, the peak in-cylinder pressure did not rise noticeably. Thus, based on these ratios of *n*-butanol injection, this section investigated the potential of optimizing combustion and lowering emissions by adjusting the pilot injection strategy of direct injection fuel. The engine's operating parameters are listed in Table 5. The main injection timing was fixed at − 12° CA ATDC. The interval between the pilot injection timing and the main injection timing was varied from 15° to 35°, while the pilot injection ratio was set between 0 and 20%. The effects of the pilot injection interval and the pilot injection ratio on the combustion and emission characteristics of the engine were investigated.

**Table 5:** Experimental conditions.

Items	Set value
Engine speed (rpm)	1800
EGR rate (%)	20
*n*-Butanol PFI ratio (%)	40
*n*-Butanol DI ratio (%)	20
Main injection timing (°CA ATDC)	− 12
Pilot injection intervals (°CA)	15, 20, 25, 30, 35
Pilot injection ratios (%)	0, 5, 10, 15, 20
Intake temperature (°C)	40
Intake pressure (MPa)	0.3

**Figure 6:**
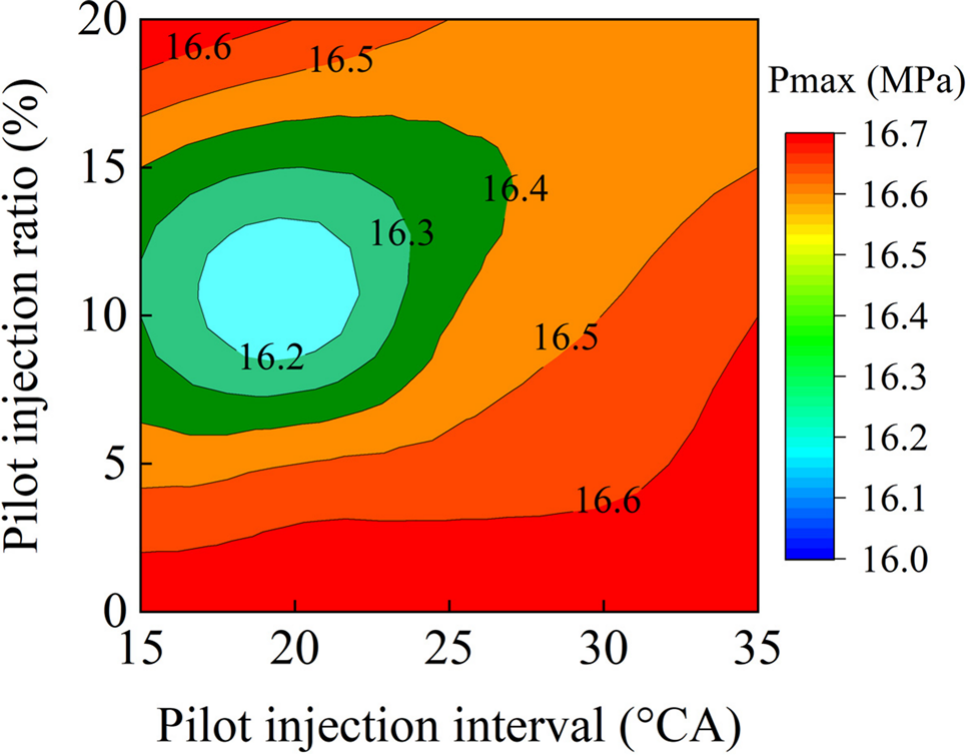
P_max_ with various pilot injection strategies.

The comparison of peak cylinder pressures for various pilot injection ratios and intervals is shown in Fig. 6. The pilot injection ratio of zero in this illustration represented a single injection. The peak in-cylinder pressure was obviously the lowest with a pilot injection interval of 20° CA and a pilot injection ratio of 10%. With increasing pilot injection ratio and keeping the same pilot injection interval, the peak in-cylinder pressure first declined and subsequently increased. With a 5% pilot injection ratio, the peak in-cylinder pressure rose when the pilot injection interval increased. By comparison, at a 20% pilot injection ratio, the peak in-cylinder pressure reduced somewhat as the pilot injection interval increased. When the pilot injection ratio was 10% and 15%, the peak in-cylinder pressure initially lowered and climbed as the pilot injection interval increased. Many active free radicals were created concurrently with exotherm when the pilot injection fuel was delivered into the cylinder at a specified pressure and temperature. The oxidation of the main injection fuel was promoted when it was injected into the active free radical zone, so the ignition delay time was shortened. The shortened ignition delay period reduced the quantity of main injection fuel for premixed combustion, resulting in a reduction in maximum cylinder pressure. However, due to the extremely short ignition delay period, the ignition timing was significantly advanced, resulting in a further rise in peak cylinder pressure. Thus, a proper pilot injection ratio and interval could limit the quantity of premixed combustion of the main injection fuel. Simultaneously, the ignition timing should not be advanced excessively. As a result, the maximum cylinder pressures could be reduced. Through the use of pilot injection, it was possible to lower the peak cylinder pressure from 16.6 to 16.2 MPa.

**Figure 7:**
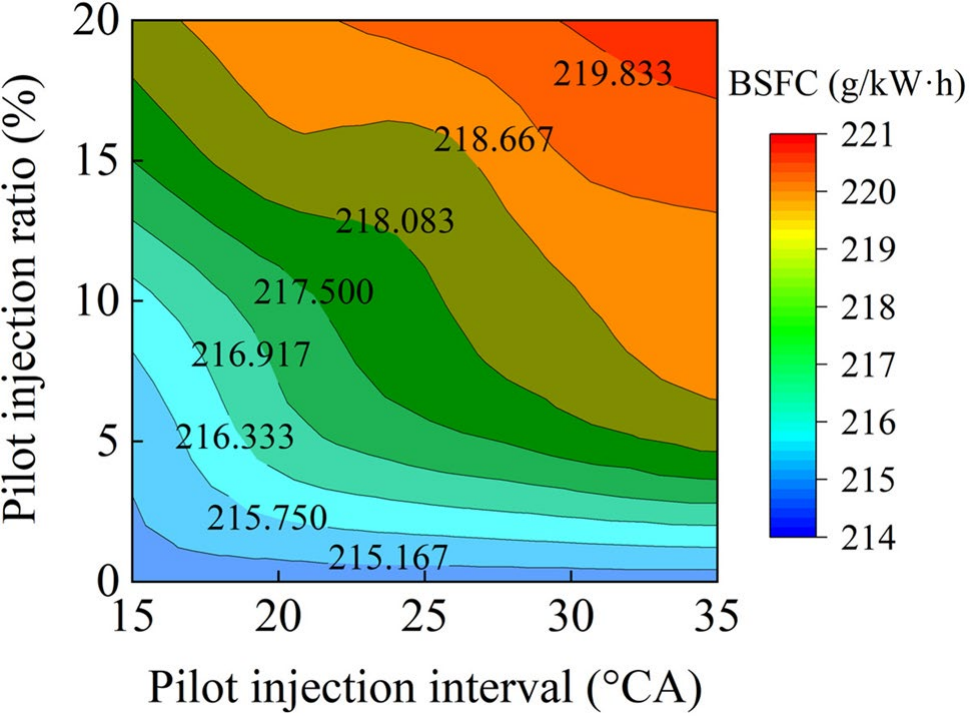
BSFCs with various pilot injection strategies.

Figure 7 compares BSFCs with a variety of pilot injection ratios and intervals. It can be noticed that BSFCs increased as the pilot injection ratio and interval rose. A higher pilot injection ratio and earlier pilot injection timing ensured that the fuel was spread uniformly throughout the cylinder, lowering the temperature and efficiency of combustion. Additionally, fuel entered the gap between the piston and the cylinder lining, resulting in incomplete combustion. An extensive pilot injection interval caused fuel to collide with the top surface of the piston or the surface of the cylinder liner wall, which contributed to incomplete combustion as well.

**Figure 8:**
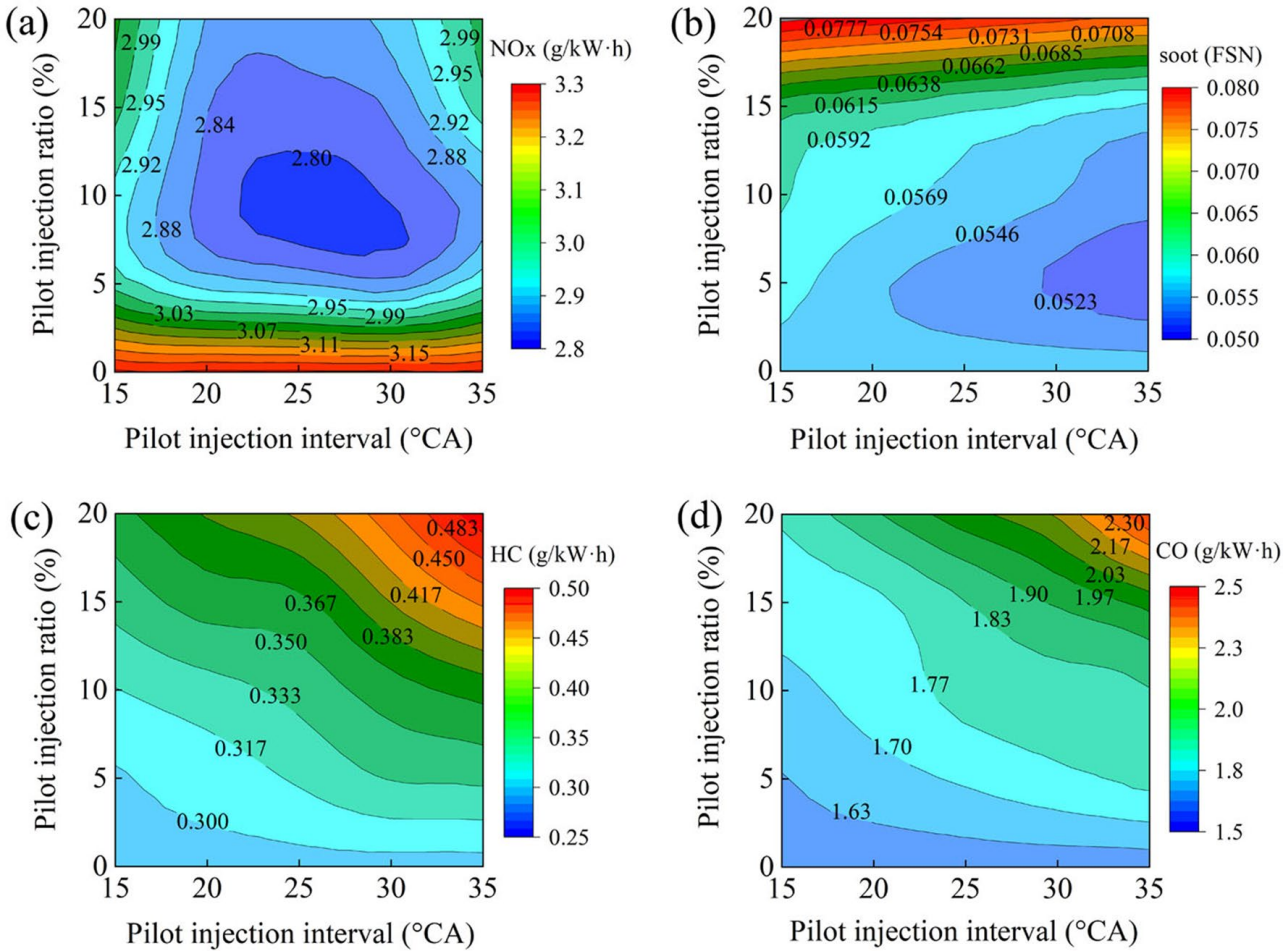
NOx emissions with various pilot injection strategies.

Figure 8a depicts NOx emissions with various pilot injection ratios and intervals. In comparison to single injection, the various pilot injection schemes significantly reduced NOx emissions. The lowest NOx emission occurred when the pilot injection interval was 25° CA and the pilot injection ratio was 10%. For a 5% pilot injection ratio, increasing the pilot injection intervals had no discernible effect on NOx emissions. NOx emissions initially dropped and then rose as the pilot injection interval increased at a 10–20% pilot injection ratio. Utilizing an appropriate pilot injection ratio and interval contributed to the reduction of NOx emissions. Due to the heat dissipation of the pilot injection fuel, if the pilot injection interval was too short, the main injection fuel would be injected into the high temperature zone, raising in-cylinder temperatures and NOx emissions. When the pilot injection interval was excessively lengthy, the ignition delay time was substantially increased, and the heat release during premix combustion increased, resulting in a rise in in-cylinder temperature and higher NOx emissions. The NOx emission could be decreased from 3.15 to 2.80 g/kW h by utilizing the pilot injection.

Soot emissions with various pilot injection ratios and intervals are shown in Fig. 8b. As the graph indicates, soot emissions rose with increasing pilot injection ratios for the same pilot injection interval. This was because the pilot injection shortened the ignition delay period of the main injection, resulting in increased diffusion combustion and soot emissions. Soot emissions decreased with increasing pilot injection intervals. As the pilot injection interval rose, the mixture became more homogeneous, decreasing soot formation. Reduced soot emissions could be accomplished by utilizing a lower pilot injection ratio and a longer pilot injection interval in comparison to a single injection.

Figure 8c shows that, in comparison to a single injection, HC emissions rose as the pilot injection interval and ratio increased. For a 5% pilot injection ratio, the HC emission at a pilot injection interval of 35° rose slightly compared to a pilot injection interval of 15°. When the pilot injection ratio was 15% and 20%, the HC emission increased dramatically as the pilot injection interval increased. With an earlier pilot injection time and a higher pilot injection ratio, the fuel mixture escaped into the gap between the cylinder and the piston, resulting in incomplete combustion and increased HC emissions. Additionally, wall wetness was caused by an early pilot injection or a high pilot injection ratio. The fuel clinging to the cylinder wall would also burn incompletely due to the low wall temperature, increasing HC emissions even more.

As illustrated in Fig. 8d, CO emissions increased with the increment of pilot injection interval and ratio compared to a single injection. The rationale for this was that with a higher pilot injection interval and ratio, the fuel was distributed more uniformly throughout the cylinder, resulting in a lower combustion temperature. Alternatively, due to the large amount of premixed combustion, the combustion speed was enhanced, leading the combustion to finish earlier, and the temperature within the cylinder decreased, resulting in the inability of CO to continue being oxidized to CO_2_. It was obvious that utilizing pilot injection would result in incomplete combustion, hence increasing CO emissions.

## Conclusions

In order to guarantee that the RCCI combustion process does not produce excessive in-cylinder pressure at high load while generating low pollutant emissions, this study proposed a novel RCCI combustion approach using port injection of *n*-butanol and in-cylinder injection of *n*-butanol/methyl oleate blend. The experimental research was conducted to investigate the combustion and emission characteristics of the engine with varied ratios of *n*-butanol PFI and DI under 90% of full load. The potential of optimizing combustion and lowering emissions by adjusting the pilot injection strategy of direct injection fuel was investigated under test conditions of R_NBPI_ = 40% and R_NBDI_ = 20%. The major conclusions are summarized as follows.

1. As the ratio of *n*-butanol PFI and DI rose, the peak cylinder pressure and heat release rate increased, the combustion duration decreased, and CO and HC emissions increased. When R_NBPI_ = 40% and 60%, NOx and soot emissions were significantly lower than R_NBPI_ = 20%.
2. Increased *n*-butanol DI ratio resulted in an increase in the peak cylinder pressure and heat release rate while lowering combustion duration. NOx and soot emissions dropped when the *n*-butanol DI ratio increased with R_NBPI_ = 20%. There was no discernible change in NOx emissions for R_NBPI_ = 40% and 60%, whereas the reduction in soot emission was modest as R_NBDI_ increases from 20 to 30%.
3. Pilot injection was feasible to lower NOx emissions while avoiding excessive in-cylinder pressure. With R_NBPI_ = 40% and R_NBDI_ = 20%, the peak in-cylinder pressure was obviously the lowest with a pilot injection interval of 20° CA and a pilot injection ratio of 10%. The lowest NOx emission occurred when the pilot injection interval was 25° CA and the pilot injection ratio was 10%. At the same pilot injection interval, soot emissions rose as the pilot injection ratios increased. CO and HC emissions rose with an increment of the pilot injection interval and ratio.

## Abbreviations

ATDC: After top dead center
BSFC: Brake specific fuel consumption
CA: Crankshaft angle
CA50: Crankshaft angle corresponding to 50% accumulative heat release rate
CO: Carbon monoxide
CO_2_: Carbon dioxide
DI: Direct injection
EGR: Exhaust gas recirculation
FAMEs: Fatty acid methyl esters
HC: Hydrocarbon
HCCI: Homogeneous charge compression ignition
IMEP: Indicated mean effective pressure
m_MO_: The mass of methyl oleate DI
m_NBDI_: The mass of *n*-butanol DI
m_NBPI_: The mass of *n*-butanol PFI
NOx: Nitrogen oxides
PCCI: Premixed charge compression ignition
Pd: Particle diameter
PFI: Port fuel injection
P_max_: Maximum pressure
PN: Particle number
PPC: Partially premixed combustion
RCCI: Reactivity controlled compression ignition
R_NBDI_: The ratio of *n*-butanol DI
R_NBPI_: The ratio of *n*-butanol PFI
